# The 10-Step Cross-cultural Equivalence Process for Developing Measures for Culturally Informed Research

**DOI:** 10.35844/001c.144211

**Published:** 2025-12-01

**Authors:** Jennifer J. Connor, Zahra Sheik, Shannon Pergament, Cawo Abdi, Crista E. Johnson-Agbakwu, Beatrice B. E. Robinson

**Affiliations:** 1 Eli Coleman Institute for Sexual and Gender Health, Department of Family Medicine and Community Health, University of Minnesota Medical School, University of Minnesota; 2 SoLaHmo, University of Massachusetts Chan Medical School, UMass Memorial Health Care; 3 Department of Sociology, University of Minnesota, University of Massachusetts Chan Medical School, UMass Memorial Health Care; 4 Collaborative in Health Equity, Obstetrics & Gynecology, Division of Preventive and Behavioral Medicine, Population and Quantitative Health Sciences, University of Massachusetts Chan Medical School, UMass Memorial Health Care

**Keywords:** Cross-Cultural Equivalence Process, Community Based Research, Translation, Female Genital Cutting, Human Sexuality

## Abstract

We describe a comprehensive ten step process for choosing and adapting existing scales or questionnaires to the Somali language for the Our Body Our Health (OBOH) study. The OBOH study aims to examine sexual pain, coping with pain, sexual health, and decision-making in a sample of Somali women living in the United States and have experienced female genital cutting (FGC). We describe how we used existing guidelines for construct equivalence, semantic equivalence, technical equivalence and translation, while also expanding on these guidelines by engaging community members through a variety of mechanisms. We also used an iterative process where decisions were made, refined, and sometimes overturned as we gathered more community driven feedback. Through this process we adapted 11 scales, created three questionnaires, and translated all. We considered five other scales and describe the reasons we did not use them for the final survey. Finally, we present lessons learned and recommendations for other researchers.

## Introduction

The Our body, Our health (ObOh) study explores sexual health in the context of female genital cutting (FGC; a.k.a. female circumcision, female genital mutilation). We investigated factors contributing to developing chronic sexual pain and other sexual dysfunctions; identified coping strategies for sexual pain management; explored sexual satisfaction and pleasure; and examined personal and treatment healthcare decision-making in Somali American women living in Minnesota, United States. Although several validated scales exist in English (the predominant language of the research team), for many of the concepts of interest to our study, we found few validated quantitative instruments available for Somali speakers. Based on our previous research (Connor et al., 2016), we expected that approximately two-thirds of our sample would need and request study instruments in Somali. Thus, we developed a comprehensive and systematic cross-cultural approach to the translation and measurement of the variables of interest in our study using a 10-step process involving adaptation, translation, and creation of several scales and items. In this manuscript, we describe our cross-cultural equivalence method highlighting the iterative instrument-creation process relating to scales and team-created questionnaires.

Epstein et al. (2015) reviewed 31 methods for addressing cross-cultural equivalence of existing scales and data collection and concluded that while no one method is necessarily best, i.e., there is no strong evidence to support one method over another. Yet, they recommend using a rigorous method as the most important factor. Components of a rigorous method includes extensive effort in translation and the use of experts (panel or focus groups).

There are many recommended approaches to translation, such as forward only, forward and back, or a team approach (Cruchinho et al., 2024). Having at least 2 translators and a reconciliation of differences process is a consistent recommendation across the various methods (Cruchinho et al., 2024). Behr and Braun (2023) found that when comparing a team approach with a simple back-translation approach to questionnaires, the team approach was preferred over the back-translation approach for minimizing problems with mistranslation, wording, accuracy, and fluency. Direct translation or word-for-word translation from one language to the other, without looking at how the words translated are used together in a sentence to form a question or an answer, can be a problem because you can lose the meaning of what you are trying to convey.

There are also variations in instructions on how to involve experts (Cruchinho et al., 2024). This may depend on the purpose, for example, whether it is to look only at semantic equivalence or if the need is broader, such as examining both conceptual and semantic equivalence. Most processes also recommend some pre-testing or pilot testing, which may include cognitive debriefing (Cruchinho et al., 2024).

For our project, we used recommendations by Flaherty et al. (1988) to guide our approach. Before data collection, they recommend a focus on: (1) content equivalence (the extent to which each item is relevant to members of the community); (2) semantic equivalence (examination of how the original meaning of each word or phrase is retained after translation); and (3) technical equivalence (choosing a data collection method that is comfortable to the participant).

## Background of the Study Population

UNICEF (2024) recently reported that communities within Somalia have made little progress in eradicating the practice of FGC; Somalia has the highest rate of FGC in the world, with approximately 98% of the female population undergoing the procedure. The World Health Organization (WHO) has created typologies of FGC (World Health Organization, 2008) Types 1–4, with subtypes under types 1–3. Type 3, also known as infibulation, is most common in Somali cultures; it is defined as “narrowing of the vaginal orifice with creation of a covering seal by cutting and appositioning the labia minora and/or the labia majora, with or without excision of the clitoris” (more specifically, the clitoral hood) (WHO, 2008, p. 10). FGC is typically performed during childhood and can have immediate and long-term health consequences (Andro et al., 2014; Kulaksiz et al., 2022; Lurie et al., 2020; Vloeberghs et al., 2012). A common outcome of FGC is sexual dysfunction, most notably, sexual pain (Bazzoun et al., 2021; Lurie et al., 2020; Pérez-López et al., 2020).

People from Somalia have migrated throughout the world in the past 30 years due to war, poverty, and climate disasters, often staying in refugee camps for decades in neighboring East African countries (Abdi, 2018; Minnesota Historical Society, n.d.; Warsame, 2001). Minnesota, with the largest Somali population in the U.S.A, has become home to over 82,000 people who identify as Somali; 77% of this population live in the Minneapolis-St. Paul metro area, and another 23% live in smaller cities or rural areas (Minnesota Compass, 2024). In planning for our study, we expected our sample of Somali women with FGC, an inclusion criterion for our study, to be born outside of the United States as FGC is illegal in the United States. The education level of many Somali people in Minnesota is low, with 44% indicating less than a high school degree (Minnesota Compass, 2024). Thus, we expected that many of our participants would fall into this lower education group. Cultural norms around family relationships, sexuality, and gender are typically different between Somalia and the U.S.A., which can result in a culture shock around these topics for Somali migrants (Abdi, 2018; Connor et al., 2016). Despite facing challenges of migrating due to difficult circumstances, the Somali community in Minnesota is vibrant and tight-knit (Areba et al., 2021). The Islamic faith is almost universal among Somali people and influences family formation, relationships, and behaviors (Yusuf, 2012). The large Minnesota Somali population allows for numerous substantial community spaces, such as Somali-owned stores, malls, restaurants, and businesses, as well as Somali charter schools. Minneapolis has been nicknamed *little Mogadishu* (Yusuf, 2012). Consequently, many in the Minnesota Somali community have retained cultural and language traditions from Somalia. Perhaps because of the large community and the recency of their migration, there is evidence migrants in Minnesota with Somali backgrounds are less acculturated than migrants from other backgrounds (Areba et al., 2021).

The cultural distance between practices and views about gender and sexuality in the Minnesota Somali refugee community and mainstream American society is substantial. The worldview, experiences, and language of the Somali population in Minnesota varies from that of North American and European researchers who have studied sexuality in predominantly white individuals with Judeo-Christian backgrounds. For these reasons, we made every effort to engage in a community-based participatory research (CBPR) approach that aimed to reach content, semantic, and technical equivalence.

## Content and Semantic Equivalence: Language

The Somali language belongs to the Cushitic language family, a branch of Afro-Asiatic languages (Warsame, 2001). The English language belongs to the Germanic family and originated in Anglo-Saxon England (Hogg & Denison, 2006). These languages are culturally and linguistically different from each other. During the process of translating information from Somali to English, cultural aspects should be considered as they play an essential role in conveying intended meaning. A balance between linguistic and cultural factors is needed during the translation process to successfully convey the original intended meaning (Behr & Braun, 2023). In addition, the Somali language has different dialects divided into three main groups; Northern/Southern, Banaadir, and Maay. When translating from English to Somali we should consider these dialects to ensure that the translation can be understood by participants taking the survey regardless of the dialect they use.

Several constructs included in our study differed in interpretation across cultures. For example, we wanted to understand emotional distress participants may experience. Yet, an assessment of emotional distress should account for cultural idioms (Im et al., 2017; Shoeb et al., 2007). Studies of pain in the Somali diaspora have found that Somali-born individuals may be more stoic when reporting pain compared to individuals born in Europe or North America (Finnström & Söderhamn, 2006; Jacobson et al., 2018). In our previous work, we found that most participants did not have a word for female orgasm (Connor et al., 2016). Thus, to achieve content and semantic equivalence, we planned to address both cultural and linguistic differences, as described further below.

## Technical Equivalence

Technical equivalence is related to finding the right data collection method for the study population (Flaherty et al., 1988). Options may include paper-pencil surveys, online surveys, in person interviews (Flaherty et al., 1988). Researchers must choose their methodology based on community comfort with the topic, reading ability, and knowledge of the technology provided. To address technical equivalence, we chose to use an audio computer-assisted self-interviewing (ACASI) method to collect survey data. ACASI allows participants to use headphones connected to a laptop computer to listen privately to instructions, questions, and responses that have been digitally recorded onto an ACASI program while corresponding text is displayed on the computer screen. This enables literate participants to read the questions while listening. Those who have low-levels of literacy can rely upon the audio component of the survey. ACASI also allows for use of graphics in visual analogue scales (VAS; for example smiley faces). Researchers who have conducted research in the Somali community have recommended the use of VAS in Likert scales to assist in accurate comprehension of each item response (Johnson-Agbakwu et al., 2016). A large body of research has demonstrated the feasibility, acceptability, and effectiveness of using ACASI with international (including African), multi-lingual (including non-English speaking), low literacy populations (Beauclair et al., 2013; Bhatnagar et al., 2013; Caldwell & Gryczynski, 2012).

ACASI has been successfully used in research studies on sexuality and FGC (Akinsulure-Smith, 2014; Phoo et al., 2022; Robinson et al., 2013). Somali cultural taboos strongly discourage sexual communication with family, friends, professionals, or strangers (Connor et al., 2016). ACASI can increase participants’ sense of privacy, thereby improving the willingness and accuracy of reporting on sensitive issues and reduce the occurrence of socially desirable responses (Dolezal et al., 2012; Estes et al., 2010; Phoo et al., 2022; Robinson et al., 2013). ACASI may mitigate concerns about sex research in the Somali community, though it also requires adapting the layout of questionnaires and response scales.

## Methods

### Overview of Cross-Cultural Equivalence Method

In this section, we describe our 10-step translation and measurement process for the translation and measurement of the variables of interest — highlighting the iterative instrument-creation process relating to scales and team-created questionnaires with the goal of optimizing reliability and validity. We present each step of our process, briefly describe measures, and provide examples of decisions made. The 10 steps were: Step 1: Identifying measures; Step 2: Panel Retreat; Step 3: Qualitative Interviews; Step 4: Integration of panel recommendations, qualitative findings, and CAB feedback; Step 5: Team review of all changes made after adaptations; Step 6: Team decisions about visual analogue imagery, practice questions; Step 7: Translation; Step 8: ACASI Pilot survey; Step 9: Final revisions prior to data collection; Step 10: Adaptation based on initial data collection.

For clarity, we first define terms used to describe team members. *Principal investigators* were the lead investigators that initiated the project. *Co-investigators* were team members who participated in the writing of the original grant, and held either faculty positions or a lead position at the community-based organization (SoLaHmo). *Community researchers* were professionals with Somali identities who were hired for the project, by either the University or the community partner; all were bilingual, had bachelor or masters degrees, and training in CBPR. Community researchers worked anywhere between 10 hours to 40 hours a week and were instrumental to ongoing feedback. *Study staff* included all of the above, in addition to the project coordinator and doctoral level data analysts. *Community members* included members of the Somali community, which included team members (co-investigator, community researchers), community advisory board members, and/or the larger Somali community. Team members may be in more than one role. For example, a Somali woman hired by the community partner may be both study staff and a community researcher. Similarly, a co-investigator of Somali descent is also a community member. Thus, we had representation of community members in all roles with the exception of the principal investigators. Community members were involved in ways that best represented their role on the project. For example, a community researcher **shaped** decisions related to language, cultural considerations, etc. throughout the process; whereas a community advisory board member **advised** the team on the selected research materials. During the planning phases, they met two to four times a year.

#### Step 1: Identifying Measures by Principal Investigators

We based our decisions about the most appropriate measures on our research questions, scientific/theoretical background of the measures, previous use of measures in a Somali sample, and the length and time it took past respondents to complete measures. Additionally, the community-academic research team collaborated to identify the most important research questions to investigate. A total of 11 measures were identified by researchers at this step (Table 1). Each measure had been validated in previous research but only two measures had been validated in the Somali language. We also reviewed medical history and genital pain questionnaires designed and used by our co-investigators in previous studies with diverse participants who had other types of genital pain (e.g., vulvodynia).

#### Step 2: Panel Retreat

We used an etic approach to adapt our measures to yield research appropriate to the local Somali culture; as noted, two measures had been translated into Somali and administered to a Somali population. Thus, they needed less review. An eight-person panel met in-person for 1 ½ days and reviewed the content of each measure (English versions) to ensure that the constructs measured were relevant and understandable to Somali American culture and that measures were translatable into Somali without losing their original meaning. The panel included both principal investigators, three co-investigators, and three community researchers. Half of the panel identified as Somali women and were bilingual; all were born outside of the U.S.A. and migrated in childhood or later to North America. The other half of the panel included White and African-American women who were born in the United States. Multiple disciplines and expertise were represented, including Marriage and Family therapy, Obstetrics and Gynecology, Social Work, Psychology, Sexual Health, Sociology, and Community Health; additionally, panelists held expertise in one or more the following: community based-research, psychometrics, qualitative research, and quantitative research. By having both Somali-born and USA-born researchers work in-person together, questions and clarifications about the intended meaning of questions were discussed and consensus reached about editing and adjusting these questions. The goal of the panel was to discuss content validity, conduct the first steps of semantic and technical validity, and choose between similar instruments. Finally, we discussed adding questions in the qualitative interview that would assist in any language or translation concerns in the final quantitative survey. The Step 2 column of Table 1 summarizes decisions made during the panel.

### Activities During Retreat.

The retreat began with a brief description of our goals for reviewing the measures, explanation of why researchers use standardized measures, and instructions for reviewing the chosen measures. The panel utilized a set of worksheets, one for each measure identified for review. Reviewing one measure at a time, first individually, and then as a group, the panel evaluated each measure. Individual items were rated as being *relevant*, *irrelevant*, or *questionably relevant* to the Somali community context. Items rated as *irrelevant* by a single team member or as *questionably relevant* by 2 or more members were reviewed for elimination; items receiving one rating of questionable relevance were reconsidered for inclusion with revisions. During this process, items were cut, edited, revised, and added to reflect appropriate cultural and theoretical concepts; examples are provided below. We also chose the “best” measure when we had more than one similar measure. For some measures, such as the Refugee Health Screener, which assessed emotional distress from a refugee context (Hollifield et al., 2013), we had few notes as everyone marked all questions as relevant. Unsurprisingly, the measures addressing sexuality elicited more discussion and notes. In Supplement 1, we have provided a sample of the compiled results for the Natsal-SF (Mitchell et al., 2012), along with notes with panel recommendations after discussion. Below, we provide examples of decisions made during the panel review.

#### Examples of Decisions Made by Panel on Scales.

##### Individual Item Revision.

The original question #4 on the CHAMP scale (Flink et al., 2015) is, “*Because of the pain, I avoid intercourse, even when I feel sexually excited.*” We revised this item to read: “*Because of the pain, I avoid intercourse*”, removing the phrase “*even when I feel sexually excited.*” We removed this phrase for two reasons – 1.) It added an unnecessary complexity to the sentence structure; and 2.) Community researchers questioned whether or not phrase “sexually excited” was translatable, calling into question semantic equivalence.

##### Individual Item Addition.

The original question # 9 on Natsal-SF: A measure of Sexual Function for Community Surveys: “*Have you sought help or advice regarding your sex life from any of the following sources (check all that apply).*” Examples of responses included family member or friend, the internet, a general practitioner or family doctor. We added the response: *“spiritual advisor or Imam”* at the panel’s suggestion.

##### Choice Between Instruments.

The principal investigators identified two sexual functioning instruments for panel review. The Natsal-SF has simply-worded questions relevant for community (not clinical) research (Mitchell et al., 2012). The Female Sexual Function Index (FSFI; Meston, 2003) is more commonly used in research, including FGC research (e.g., Catania et al., 2007). The panel thought the majority of FSFI questions would be difficult or impossible to translate. As an example, comparing the two scales on questions about arousal, one can readily see the difference. The Natsal uses the same stem for sexual problem questions: “have you experienced any of the following for a period of 3 months or longer”;”no excitement or arousal during sex. The FSFI asks eight questions about arousal, including items with cultural idioms — “over the past 4 weeks, how would you rate your level of sexual arousal (“turned on”) during sexual activity or intercourse;” and questions that are very similar to one another with small nuanced differences — “over the past four weeks, how often did you maintain your lubrication (“wetness”) until completion of sexual activity or intercourse” and “over the past four weeks, how difficult was it to maintain your lubrication (“wetness”) until completion of sexual activity or intercourse.”

##### Choice to Remove Instruments.

The Vulvar Pain Assessment Questionnaire Inventory (VPAQI; Dargie et al., 2016) is an 18-item inventory assessing vulvar pain and its impact, created for vulvodynia patients. Some questions were not relevant to women with FGC. Many questions had sub-questions, which made the measure quite lengthy (e.g., *“How much does your vulvar pain negatively interfere with the following:”* – 11 activities are listed, each requiring an answer. We decided not to use this particular inventory due to length.

##### Suggestions for Additional Instruments.

The panel recommended that we add an instrument assessing the couple’s relationship due to the importance of relationships when studying sexuality.

#### Step 3: Qualitative Interviews

We used qualitative interviews to gain a richer understanding of our subject matter to: (1) gather data about Somali women’s perspectives on sexuality, coping with pain, and interventions; these results were published elsewhere (Connor et al., 2023, 2024); and (2) guide quantitative survey development. Examples of questions we used for this latter purpose included:

- What words would you use to describe how sexual pain felt?
- Was/is there anything that you did to minimize pain that worked well for you?
- In the future, we plan to ask women about sexual satisfaction or the feeling of being happy with their sex life. We are looking for the best ways or words to describe that feeling. What words come to mind when I say feeling happiness in your sex life?
- What are some good words in Somali to describe a female orgasm?

##### Examples of Qualitative Research Informing ACASI Survey Development.

Our qualitative results proved helpful in developing items for our ACASI survey. Participants described frequently used words describing sexual function, pain, and satisfaction, and used these suggested words in the final survey. For example, many participants described the word “cutting” to describe pain; thus, we included this word in the pain section of the survey. Participants emphasized support from their partners as important to minimizing sexual pain (Connor et al., 2023); thus, we added questions about partner support and behavior. Though the panel was concerned that the term “*sexual satisfaction*” might be unclear, participants indicated that the phrase *sexual satisfaction* was appropriate and understandable; we retained this phrase in the survey. We learned that many participants understood the phrase “*biyo bax*” to mean female orgasm (Connor et al., 2023); we used this term in our survey as well.

Frequently, participants spoke about medical concerns they thought were caused by FGC and were alleviated by deinfibulation. Thus, we added questions about menstruation and urinary problems as it was clear that community members felt these were important outcomes. Qualitative findings were helpful in guiding survey questions about FGC and deinfibulation history. For example, several participants reported they were deinfibulated more than once (e.g., if physician did not sew open the tissues after a vaginal birth); thus, we asked questions about being deinfibulated more than once.

Before conducting the qualitative interviews, we created watercolor images of vulvas representing different types of FGC. We used the data collected in the interviews to demonstrate that adding illustrations to self-report questions about FGC type provided clarity and specificity to these types of questions (Chaisson et al., 2023; Johnson-Agbakwu et al., 2023). We used these illustrations in the final ACASI survey.

#### Step 4: Integration of Panel, CAB, and Team Recommendations and Qualitative Findings

Based on feedback from the panel, the community advisory board, and team members, as well as findings from the qualitative data, we integrated this feedback into our measurement decisions (Table 1, Step 4). “Small edits” referred to minor changes, e.g., using synonyms for original words based on semantic equivalence or cultural appropriateness. Similar to Step 1, we prioritized adding measures with low burden and those already used in a Somali sample. For example, Perovic et al. (2021) published a manuscript using the *McGill Pain Inventory* – Short Form (Melzack, 1987) in a Somali-Canadian sample; we requested and received the translated version for use in our study. Additionally, we asked urologists who provide care to Somali patients in our medical school to review several female lower urinary tract symptom questionnaires which they deemed too complicated. Thus, they created a simplified version of existing scales, which reflected their clinical experience.

We removed two scales: *Short form of the Posttraumatic Growth Inventory* (PTGI-SF; Cann et al., 2010) and the *West Haven Yale Multidimensional Pain Inventory, Social Support Subscale* (WHYMPI; Kerns et al., 1985). The PTGI-SF asked participants about personal growth in the face of challenges, with multiple items addressing values and faith (e.g., “I have a greater appreciation for the value of my own life”). Community researchers advised their removal given that in Islam, having great appreciation for the value of one’s life is assumed and never questioned. They also doubted whether all participants viewed FGC as a traumatic event; thus the scale might not work as intended. Additionally, we determined that the WHYMPI became redundant after adding the relationship satisfaction survey.

We created or adapted sections of the survey. Our inter-disciplinary team had team members with expertise to write, edit, and comment on previously created items, such as medical background and demographic items. Two physicians with extensive experience in delivering care to the Somali community provided detailed feedback on all medical questions. We sought input from colleagues with expertise in measurement development and community-based research when adding measures and creating our own items (e.g., demographic items). We incorporated feedback about language from CAB meetings, e.g., use “husband/partner” instead of “sexual partner” and “sex” instead of “vaginal intercourse” or other similar phrases.

Several investigators suggested we capture experiences of discrimination and/or racism within the healthcare system because of their impact on health (Williams et al., 2019). We brought this decision to the CAB, both in 2019 and again after George Floyd’s murder in 2020. The CAB discouraged this, worried that this measure would be perceived by participants as too negative and not directly related to our stated purpose; they noted that our study emphasis, FGC and pain, was already focused on negative experiences. CAB members encouraged us to add items assessing participant assets and resilience. Their feedback led to our decision to avoid adding items on discrimination/racism and led us to be vigilant about the possible perception that we only are interested in negative experiences within the Somali community. The decision to not use this scale was difficult, but we were most influenced by the strength of the reaction by the CAB. The CAB members had all voiced support of the overall project. Yet, amongst this group of supporters, all voiced concern about including a measure that could create a sense of mistrust between community and researchers. As one of the primary roles of the CAB is to help prevent any missteps in community trust, we chose to follow their recommendation.

#### Step 5: Team Review of All Changes Made After Adaptations

At Step 5, the team, including the community researchers, focused on clarity (e.g., instructions) and details of the overall survey. We made edits to ensure consistency across questions to reduce survey burden.

#### Step 6: Team Decisions About Visual Analogue Imagery, Practice Questions, etc.

Based on panel input and research literature (Blum et al., 2014), we added visual analogue imagery as much as possible to improve comprehension of item response options (e.g., faces accompanying pain ratings). We wrote various practice questions designed to orient and train participants in the use of the ACASI survey on a talking computer. Based on community researcher preferences, we decided to use questions about the common food sambusas – understandable to all Somali people – to practice the several types of questions in ACASI. The practice questions were:

- Do you like sambusas? (yes/no)
- How many sambusas have you had today? (type in a number)
- How often do you eat sambusas? (frequencies broken into categories)
- What filling do you like? (multiple response options, choose up to three)
- How much do you enjoy eating sambusas (never, sometimes, always)
- I know how to make sambusas (5-point Likert scale)

#### Step 7: Translation

All scales were translated into 5th–6th grade reading level Somali by a professional Somali translator, except the three scales already translated and validated by other researchers (Table 1, Step 7). The professional translator focused on meaning for meaning rather than word-for-word translation. This process promoted conceptual equivalence over literal linguistic equivalence (Behr & Braun, 2023; Hollifield et al., 2013; Johnson-Agbakwu et al., 2016). Two bilingual community researchers on the project reviewed these translations and used an iterative process to revise and reach consensus on all items. For example, they made edits to simplify and reflect everyday Somali language used by the Somali population in Minnesota. The translated measures were then reviewed by bi-lingual/bi-cultural community members on our Community Advisory Board who did not participate in the translation process, including the 3 instruments already adapted in prior research for use with Somali people

#### Step 8: ACASI Pilot Survey

Before the pilot, community researchers recorded the Somali version of the survey into ACASI. We used the computer-generated ACASI voice for the English version of the survey. Pilot testing took place in two phases. During the first phase, six study staff completed the English survey in ACASI and two community researchers completed the Somali survey in ACASI. Study staff provided feedback on grammatical and technical errors; clarity of instructions, questions, and response options; and sound quality. During the second phase, three CAB members completed both the English and Somali version(s) of the survey in ACASI; CAB members provided similar feedback as those in the first phase and reflected on any concerns about appropriateness of items (Table 1, Step 8).

#### Examples of Decisions Made After Pilot Testing.

##### First Phase.

Pilot reviewers provided feedback from their expertise and cultural backgrounds. For example, one question about vaginal douching modeled after a vulvodynia research questionnaire and included as a possible covariate due to possible associations between douching and genital infections (e.g., Yıldırım et al., 2020), prompted disparate and conflicting feedback. We defined douching as, *“a method to wash out the vagina with water and vinegar.”* One investigator, a faculty member and a Somali community member noted, “douching is not common. Do we care about this? Possible question to cut.” Another investigator, a faculty member with European ancestral background queried, “are we accidentally implying it’s a good thing or …. encouraging it?” A community researcher recommended that we “change the definition. Some people might not use vinegar and only water.” This feedback raised questions about the clarity and relevance of the question; thus, we decided not to use it.

Feedback from community researchers led us to use our Somali staff to record the English version of the survey, rather than using the computer-generated voice. For example, even in English, Somali individuals commonly use the words “Sunna” and “Pharaonic” to describe FGC types; thus, we used these words in the English version of the survey. However, the computer voice always mispronounced them.

##### Second Phase.

CAB members generally liked the survey layout, audio, and content, but gave us negative feedback regarding two scales: the Short-form McGill Pain Questionnaire and the Female Genital Image Scale (Herbenick & Reece, 2010). They, along with several study staff, thought the Short-form McGill Pain Questionnaire was too long and difficult to answer. We decided to reduce the measure from 24 items to five pain qualities that came up in the qualitative interviews (i.e., throbbing, sharp, hot-burning, splitting, itching). Though having all 24 items may have provided valuable information about types of pain, it seemed that many of these nuanced similar items would confuse and annoy our participants. One CAB member thought participants would be offended by the Female Genital Image Scale. As genital image was not central to our study aims, we removed it.

#### Step 9: Final Revisions Prior to Data Collection

We incorporated feedback from the pilot interviews and finalized the survey. Once finalized, we began data collection.

#### Step 10: Adaptation Based on Initial Data Collection

Within the first weeks, data collectors noticed that many participants were confused by the deinfibulation history and shared decision-making part of the survey and asked for help throughout this section. Data collectors suggested we change the format of this section from ACASI survey to an interview. Much of the participant confusion arose from the need to adapt the question based on the previous answer (e.g., if a participant stated she had been deinfibulated before she was married, she would then receive questions about her decision to be deinfibulated at that point in time). Once data collectors used the interview format, they used the exact words the participant used to refer to the decision she had made; this alleviated most misunderstandings.

## Lessons and Recommendations

Throughout the implementation of the 10 cross-cultural equivalency steps, our belief that a community-academic partnership would be invaluable in developing measures and instructions that were relevant, clear, well-translated, and appropriate was confirmed. Unexpectedly, delays in data collection due to COVID-19 gave us additional time for collaborative engagement in reviewing and refining our survey; time well spent and time that we would recommend other researchers build into their planning when conducting cross-cultural research.

As we discussed in our introduction, there are several methods for adapting measures for different ethnic and cultural groups. Some of the more common recommendations include integrating community feedback to ensure content and semantic equivalence, pilot testing, and using a data collection method best fitting the population (Epstein et al., 2015; Flaherty et al., 1988). Multiple guidelines for the translation and validation of translated measures exist to help researchers determine if measures need adaptation for the study population and context (Behr & Braun, 2023; Pan & de La Puente, 2005). At times, guidelines may be contradictory; thus, decisions may require additional deliberation. Our study focused on stigmatized and taboo topics around sexuality and FGC in a migrant population. Many study variables had a high potential for varied meanings in different population groups (e.g., sexual function, sexual satisfaction, emotional distress). Additionally, our participants were born in East Africa, which has a low literacy rate (A. A. Mohamed & Aiayanna, 2013). Historically, people from Somalia relied on oral traditions to convey knowledge, with less formalized education in reading and writing (Warsame, 2001; William Johnson, 2006). Thus, we had many considerations beyond just word-for-word translation to contend with. We advise research teams working with similar contexts to consider the following lessons learned and recommendations.

### Lessons Learned and Recommendations

#### Recommendation 1

##### Involve community members (researchers and lay members) immediately and throughout the entire process — as research partners, employees and community advisory board members.

Community members can advise on language, appropriateness, relevance, visual imagery, and interpretation of any qualitative findings that will be used for survey development. They can also assist in piloting surveys and technology.

#### Recommendation 2

##### Conduct a panel review to finalize survey decisions.

Panel members should include bilingual community members (who may or may not also be academics), experts in survey methods and measurement, in CBPR, in the research topic being studies, and in health research, including clinicians who treat patients from the community.

#### Recommendation 3

##### Conduct the panel review (Table 1, Step 2) after team building and completion of the qualitative interviews.

This recommendation is based on two experiences. First, community researchers that had only been working on the project for two months did not raise issues on the panel that they later brought forward. New employees may have felt intimidated by being on a panel with individuals with MD’s, PhD’s, and more experienced researchers. Had we waited to establish greater rapport across the community-academic team first, there may have been more frank discussions on key measures that later required adaptation.

Second, completing the analysis of the qualitative study data after convening the panel led to missed opportunities to review some measures (Table 1, Measure). A main finding of the qualitative study was that participants viewed the support of their spouse as key in reducing their sexual pain early in the relationship and preventing chronic sexual pain later on (Connor et al., 2023). The study team added a relationship satisfaction measure to assess spousal support at Step 4, well after the panel met. Thus, the panel was not able to review this added measure. Ideally, qualitative data should be collected before measures are reviewed and finalized so that all measures used in your study be reviewed by the panel. However, with this change it becomes even more important that the CAB also review and help draft the qualitative questions prior to data collection.

#### Recommendation 4

##### Develop a means for evaluating community input that is at odds with other scientific considerations.

For example, community members found the language on the FSFI to be difficult to comprehend and too clinical, and other researchers have noted limitations in using the FSFI in Somali populations (Catania et al., 2007). Therefore, we used two other measures assessing sexual functioning instead (Natsal, CSFQ). However, the FSFI has become the most commonly used measure of sexual function in studies examining the impact of FGC on sexuality, partially because the FSFI has been validated in Arabic (Abdalla & Galea, 2019; Abdelhafeez et al., 2020; Dura et al., 2024; Eftekhar et al., 2019; Hassannezhad et al., 2024; A. H. Mohamed et al., 2022; Mustafa et al., 2019; Obaid et al., 2019; Paslakis et al., 2020; Rouzi et al., 2017; Sedik et al., 2021; Shafaati Laleh et al., 2022). Between 2019 and July 2024, at least 12 published studies have used the FSFI in FGC research. Not using the FSFI made it difficult to compare our results to the results in other studies and led us to sometimes question this decision. A community-based approach highlights differences between the preferred choice in the overall academic literature and the best choice for a given language and/or culture. We ultimately concluded that we made a good choice, as it is critical that the question make sense to the participant in order for the data to be valid and useful. It is possible that the FSFI works well in some communities, but not all. We suggest that future research compare the FSFI to other measures preferred by community members to determine which measures seem to work better in certain populations and for certain sexual problems.

#### Recommendation 5

##### Utilize qualitative findings to inform decisions about existing measures or development of new measures.

In particular, we found it effective to ask about words that may not exist in the language of the participant and compile participants’ descriptions of the construct being addressed.

#### Recommendation 6

##### Understand the language(s) of the original measures and translated measures.

Most importantly, take into account: (a) words from the original measure that do not have an equivalent word; (b) how specific cultures impact the understanding of a word, for example, what does the word “sex” mean in Somali; (c) common dialects within the region of your study population and how they may differ from each other and the translator’s dialect.

#### Recommendation 7

##### Utilize visual analogue response scales with culturally appropriate imagery.

Community members had extensive conversations about the appropriate images and colors to use in ACASI response scales, thus helping us select the most culturally appropriate and well-defined scales.

#### Recommendation 8

##### Invest in pilot studies.

CAB members provided invaluable feedback during the pilot study. They assisted in identifying questions and sections that resulted in survey fatigue, provided language and translation suggestions, and provided reassurance that the majority of the survey was user-friendly and culturally appropriate. We found the pilot study process well worth the considerable time and effort.

#### Recommendation 9

##### Use human voices for ACASI audio files.

For efficiency, we originally planned to use the computer-generated English voice to read the questions to participants in ACASI. As noted earlier, the CAB and community researchers felt that the computer-generated English voice frequently had poor pronunciation of Somali words used in the English version (e.g., Sunna), which was off-putting. In addition, we realized that by having a local Somali woman’s voice read the questions in both the English and Somali versions, we removed a possible methodological confound between the English and Somali versions of the ACASI survey.

#### Recommendation 10

##### Be flexible about revising data collection early in the study if problems arise.

Two main reasons we chose ACASI were privacy and reducing social desirability. Data collectors informed the study team that participants were frequently asking for help with the shared decision-making portion of the survey. By responding to this problem and developing a brief a face-to-face interview to collect data in this area, we increased our confidence in the validity of our data. We recommend that future researchers solicit this type of feedback from data collectors, and to consider combining face-to-face interviews and ACASI for complex surveys. This method allowed us to use an interview for a section that required flexible questions (i.e., to match the exact wording of each participant’s response), while retaining ACASI for more sensitive questions about sexual behavior.

## Conclusion

We conducted a lengthy and systematic process to adapt measures and data collection methods from English to fit our Somali respondents. We argue that this process strengthened our study and enhanced confidence in our findings. The COVID pandemic occurred soon after we collected our qualitative data and before we started quantitative data collection. This resulted in increased time to integrate the qualitative findings into measure adaptation, additional conversations with community researchers on survey development, and extra time to record audio versions of the survey in ACASI. We believe this additional adaptation time with community members improved our survey; at the very least, it increased our confidence that the survey was understandable to, and appropriate for, participants. This process may not always be feasible, but we recommend that researchers make efforts to put more time than they think they need in adapting measures for cross-cultural research. We also argue that, in the end, this process may help reduce time spent later in adaptations of measures and produce meaningful results.

## Supplementary Material

Supplement 1

## Figures and Tables

**Table 1.** Scale/Measure Section Description and Decisions

Scale/Measure	Construct	Step 1	Step 2	Step 4	Step 7	Step 8
Natsal Sexual Function Mitchell et al (2012)	Sexual Function	Included	Reviewed	Small edits	Translated	No changes after pilot
CHAMP Sexual Pain Coping Scale Flink et al (2015)	Coping with sexual pain	Included	Reviewed	**Added items about coping with sexual pain to reflect qualitative results and community feedback** (not to be included in what is considered the CHAMP scale scoring; new items for same construct).	Translated	No changes after pilot
The Decisional Conflict Scale O’Connor, 1993	Shared decision making	Included	Reviewed	**Team created deinfibulation decision items**	Translated	No changes after pilot
Avoidance-Endurance Questionnaire Hasenbring et al., 2009	Pain response patterns	Included	Reviewed – many items not relevant to sexual pain; decided to revise	**Revisions to include sexual pain instead of generic pain**	Translated	No changes after pilot
Vulvar Pain Assessment Questionnaire Inventory Dargie et al., 2016	Pain, coping, impact of pain	Included	Reviewed – too long and not always relevant, **removed**			
Female Sexual Function Index Wiegel et al., 2005	Sexual Function	Included	Reviewed – not translatable, **removed**			
Refugee Health Screener Hollifield et al., 2013	Depression, anxiety, trauma	Included	Reviewed	Small edits	Reviewed existing translation	No changes after pilot
Bicultural Involvement Questionnaire Johnson-Agbakwu et al., 2016	Acculturation	Included	Reviewed	Small edits	Reviewed existing translation	No changes after pilot
Short form of the Posttraumatic Growth Inventory Cann et al., 2010	Benefit finding	Included	Reviewed	**Removed**		
West Haven Yale Multidimensional Pain Inventory Kerns et al., 1985	Social Support	Included	Reviewed	**Removed** – redundant		
Genital Self-Image Scale Herbenick & Reece, 2010	Genital self-image	Included	Reviewed	No changes	Translated	**Removed** based on feedback
Medical Questions – Harlow vulvodynia study Harlow et al., 2009	Medical history	Included	Reviewed	Revised and **added items relevant to FGC**	Translated	Small edits
FGC History Questions	FGC history			**Created** by team members	Translated	No changes after pilot
Kansas Marital Satisfaction Scale Schumm et al., 1983	Relationship satisfaction			**Added** based on panel feedback	Translated	No changes after pilot
Changes in Sexual Function Questionnaire Keller et al., 2006	Sexual function			**Added** based on qualitative findings	Translated	No changes after pilot
McGill Pain Inventory Short form Melzack, 1987	Pain intensity			**Added** based on most recent research in Somali community	Reviewed existing translation	Removed several items – survey burden
Lower Urinary Tract Symptoms	Urination Problems			**Created** by team members	Translated	No changes after pilot
Demographics	Demographics			**Created** by team members	Translated	No changes after pilot

Step 1: investigator identified measure; Step 2: panel retreat; Step 4: integration of panel recommendations, qualitative findings, and CAB feedback; Step 7: translation; bolded items are major action steps.
