# Supplementary material for: The 10-Step Cross-cultural Equivalence Process for Developing Measures for Culturally Informed Research
Source: J Particip Res Methods. Author manuscript; Available in PMC 2026 May 30. (PMC13221084; doi:10.35844/001c.144211)
Supplement: Supplement 1 [file NIHMS2168795-supplement-Supplement_1.pdf]

The Natsal-Sexual Function Summary Scores

**Item**	**Yes (Relevant)**	**Questionable**	**No (Not Relevant)**	**Notes**
Q1. In the last year, have you experienced any of the following or a period of 3 months or longer?				
1. Lacked interest in having sex	6[1]			
2. Lacked enjoyment in sex	6			
3. Felt anxious during sex	6			
4. Felt physical pain as a result of sex	6			
5. Felt no excitement or arousal during sex	6			
6. Did not reach a climax (experience an orgasm) or took a long time to reach a climax despite feeling excited/aroused	3		1	Needs clarification – ask about wording in qualitative interviews and revise.
7. Had an uncomfortably dry vagina (asked of women only)	5			Needs clarification – ask about wording in qualitative interviews and revise.
Q2. My partner and I share about the same level of interest in having sex	6			
Q3. My partner and I share the same sexual likes and dislikes	4	2		Some question as to whether or not partners discuss likes and dislikes; deferred decision
Q4. My partner has experienced sexual difficulties in the last year	3			
Q5. I feel emotionally close to my partner when we have sex together	7			
Q6. Overall, I feel satisfied with my sex life	7			
Q7. I feel distressed or worried about my sex life	7			

[1] At times, people chose to discuss and did not provide a written suggestion. Thus, when fewer than 8 people rated an item, the remaining were unsure.

Item	Yes (Relevant)	Questionable	No (Not Relevant)	Notes
Q8. I have avoided sex because of sexual difficulties, either my own or those of my partner	7			
Q9. Have you sought help or advice regarding your sex life from any of the following sources in the last year?	7			
1. Family member/friend	6	1		
2. Information and support sites on the internet	6	1		
3. Self-help books/Information leaflets	7			
4. Self-help groups	7			
5. Helpline	7			
6. GP/Family doctor	3	3	1	
7. Sexual health/GUM/STI clinic	2	5		GUM is UK language; change to OBGYN and retain
8. Psychiatrist or psychologist	5	2		
9. Relationship counsellor	6	1		
10. Other type of clinic or doctor	4	1		
11. Have not sought any help	4	2		
				Add Spiritual Advisor

Mitchell, K. R., Ploubidis, G. B., Datta, J., & Wellings, K. (2012). The Natsal-SF: A validated measure of sexual function for use in community surveys. *European Journal of Epidemiology*, *27*(6), 409–418. https://doi.org/10.1007/s10654-012-9697-3
